# Recent Progress in Wireless Sensors for Wearable Electronics

**DOI:** 10.3390/s19204353

**Published:** 2019-10-09

**Authors:** Young-Geun Park, Sangil Lee, Jang-Ung Park

**Affiliations:** 1 Nano Science Technology Institute, Department of Materials Science and Engineering, Yonsei University, Seoul 03722, Korea; younggeun@wearablelab.net (Y.-G.P.); sangil@wearablelab.net (S.L.); 2 Center for Nanomedicine, Institute for Basic Science (IBS), Seoul 03722, Korea

**Keywords:** wireless sensors, wearable electronics, electronic skins, smart contact lenses, neural interfaces, retinal prostheses

## Abstract

The development of wearable electronics has emphasized user-comfort, convenience, security, and improved medical functionality. Several previous research studies transformed various types of sensors into a wearable form to more closely monitor body signals and enable real-time, continuous sensing. In order to realize these wearable sensing platforms, it is essential to integrate wireless power supplies and data communication systems with the wearable sensors. This review article discusses recent progress in wireless technologies and various types of wearable sensors. Also, state-of-the-art research related to the application of wearable sensor systems with wireless functionality is discussed, including electronic skin, smart contact lenses, neural interfaces, and retinal prostheses. Current challenges and prospects of wireless sensor systems are discussed.

## 1. Introduction

Over the past several decades, the use of electronics in a wearable form to monitor body signals and the surrounding environment has led to major developments in healthcare and environmental sensors [1,2,3,4,5]. A wide range of wearable sensing technologies is commercially available, such as smartwatches that monitor heart rate and armbands that monitor gesture control [6,7]. Until now, commercial wearable sensors have forms that add sensor functions to existing portable electronic systems with rigid forms. Therefore, numerous research of wearable sensing systems has shown that they can intimately contact the surface of body parts or be implanted into the body with minimal damage to biological tissue; examples include stretchable electronic skin [8], soft neural interfaces with tissue-level stiffness [9], and smart contact lenses fabricated on the soft contact lens materials for minimal eye irritation [10]. In addition to the development of stretchable or transparent wearable sensors that minimize discomfort to users [11,12,13,14], one of the main research goals of wearable sensing devices is the continuous detection of biological signals while being attached or implanted into the user’s body. Wireless technologies that provide remote power or data communication play an essential role in wearable sensing systems. For instance, when conducting ocular diagnostics via smart contact lenses, if a wire extends from the lens to the external analysis instrument, the user may be unable to blink comfortably and must remain tethered to the instrument, which prevents the user from performing daily activities while continuous sensing is happening. In the case of implanted wearable devices, such as neural interfaces that continuously monitor electrical activity of the brain during the day, these devices should be able to wirelessly transmit internal brain signals to external measuring devices [3,15].

As sensors require wireless functions, two approaches for the development of sensor systems have evolved: (1) integration of wireless data or power transferable circuits in the conventional sensors and (2) development of sensors that detect body signals by measuring radiofrequency. The first approach is, for example, sending signals to a smartphone by connecting the measured drain current of transistor-type pressure sensors to a Bluetooth module [16,17], and it can also be applied to other kinds of sensors. The second approach is a passive sensing approach. For example, the development of split-ring resonator-type pressure sensors in which the resonance frequency of the split-ring resonator changes due to pressure [18,19], and it can be used for many other types of sensors with a variety of formats. Both approaches have advantages and disadvantages, but by properly combining these wireless approaches, a sensor system optimized for a variety of applications in wearable electronics can be designed.

**Figure 1:**
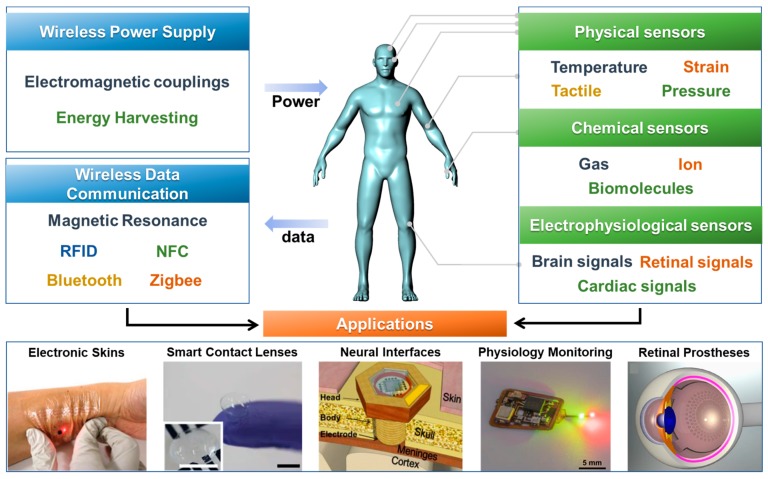
Components in wireless, wearable sensor systems and their representative applications.

In this review, the wireless power supply and data communication technologies for wearable sensor systems are introduced. And then, wireless sensors are discussed, including physical, chemical, physiological sensors. Next, the wearable sensing systems with wireless functions are discussed as device applications such as electronic skins, smart contact lenses, neural interfaces, physiological monitoring devices, and retinal prostheses. The contents of this review are summarized in Figure 1. Finally, the advantages, current challenges, and perspectives regarding the direction of further developments in wearable sensor systems are also discussed in Prospects and Conclusions section.

## 2. Wireless Technologies for Sensors Systems

In this section, wireless power supply and data communication technologies are introduced with their characteristics. Also, their advantages and weaknesses are compared.

### 2.1. Wireless Power Supply

For wireless sensors to operate in a sustainable manner, an approach that can provide continuous power is needed. Several studies of wireless wearable sensors reported that batteries can be used to solve wired power supply problems, but it is difficult to use batteries without power transfer technologies, in that case battery replacement is not the desired process in certain applications, such as implanted devices. Therefore, wireless power supplies are an essential technology for the successful implementation of various types of wireless wearable sensors.

#### 2.1.1. Wireless Power Transfer

Wireless power transfer uses electromagnetic fields to transfer power between a pair of antennas. Various kinds of electromagnetic power transfer can be used for data transmission; thus, it has been widely used as a solution for supplying wireless power to sensors. Radiofrequency (RF) power is transferred by inductive electromagnetic coupling of a transmitting antenna connected to an external power source to a receiving antenna. The delivered power is used to charge the battery or to directly operate the wearable electronic device as a battery-less system [20]. The size of the device system without a battery can be miniaturized and the lack of batteries solves certain safety issues, such as battery explosion or electrolyte leakage. However, if the application requires a large amount or continuous power for data transmission, it is reasonable to integrate batteries together [21].

**Figure 2:**
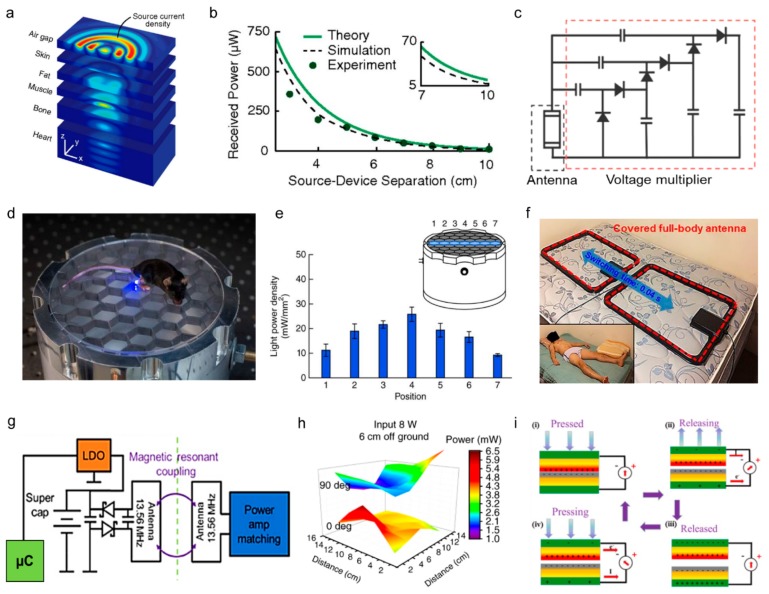
Wireless power supply technologies for wearable sensors. (**a**) Expanded view of the magnetic field in tissue multilayers, revealing propagating waves that converge on the coil (linear scale) (Reproduced with permission [24]). (**b**) Theoretical, numerically simulated, and measured power received by a 2-mm diameter coil as a function of distance when coupling 500 mW into the tissue (Reproduced with permission [24]). (**c**) Circuit diagram of wireless power receiver with voltage amplifier (Reproduced with permission [25]. Copyright 2015, John Wiley and Sons). (**d**) Resonant cavity powers a wireless device in a mouse on the surface of the cavity (Reproduced with permission [26]. Copyright 2015, Springer Nature). (**e**) Calculated light power density across the width of the behavioral area above the resonant cavity (Reproduced with permission [26]. Copyright 2015, Springer Nature). (**f**) Photograph of dual-antenna system configured for full-body readout on a mattress, with inset of a subject lying on top of a ~5-cm-thick pad that covers the antennas. Subject: 27 years of age, male, 90 kg (Reproduced with permission [27]. Copyright 2018, American Association for the Advancement of Science). (**g**) Block diagram of the electrical working principles. LDO, low-dropout regulator; µC, microcontroller (Reproduced with permission [28]. Copyright 2018, Springer Nature). (**h**) The optical output intensity of a regulated implant at 3 and 9 cm height in a single primary antenna (power 8 W in a 30 cm × 30 cm cage). a.u., arbitrary units (Reproduced with permission [28]. Copyright 2018, Springer Nature). (**i**) Electricity generation mechanism of the contact-separation TENG (Reproduced with permission [29]. Copyright 2019, Elsevier).

During inductive coupling, when an alternating current is applied to the transmitting antenna, an oscillating magnetic field is formed, and electricity is induced by the inductance of the receiving antenna when the magnetic field from the transmitting antenna is shared with the receiving antenna. The mutual inductance between the two antennas is expressed as [22]:

$$M=k\sqrt{L_{1}L_{2}}$$

where k is a coupling coefficient that includes the term of the distance between two antennas and the permittivity, *L_1_* and *L_2_,* is the inductances of the transmitting and receiving antenna. For effective inductive coupling, the high inductance of the antennas and a short distance between the two antennas are required. The inductive coupling has been widely used as a wireless power supply for wearable electronics since it represents a non-radiative wireless power transfer technology, which is compatible with being used on or inside the body [21,23]. Ho et al. demonstrated wireless power transmission from the antenna at the porcine chest wall to electronic devices implanted in the heart surface (Figure 2a). When coupling 500 mW into the tissue, the antenna inside the body received approximately 200 µW, in the case of a 4 cm separation between the transmitting and receiving antenna (Figure 2b) [24]. Kim et al. connected the receiver antenna and voltage multiplier (Figure 2c) to a supercapacitor to operate a strain sensor. In this system, the power transfer efficiency was approximately 66%, and 1.5 V could be supplied to the supercapacitor at a maximum distance of 70 cm from the power source. By the inductive coupling of 0.4 W input power, a supercapacitor was charged, and the supercapacitor supplied constant 70 µA to the strain sensors for 35 seconds [25]. 

Many battery-free sensor systems use magnetic fields arranged in the surrounding environment as a continuous power supply. Montgomery et al. fabricated a 21 cm-diameter resonant cavity and placed a mouse on the cavity so that an implanted device in the mouse could operate wherever the mouse moved in the cavity, as shown in Figure 2d. When the input power was 3.2 W, more than 10 mW was received by the receiving antenna in the implanted devices (Figure 2e). The power capability of the antenna is varied across the cavity, from 5.6 to 15.7 mW [26]. Use of a large transmitting antenna allows the electromagnetic field environment to be scalable. Han et al. formed two rectangular coil antennas on a bed mattress, with an antenna dimension of 80 cm x 56 cm, and used this as a transmitting antenna that can operate 65 sensors attached to the entire body, as shown in Figure 2f. When the transmitting antenna was driven by 12 W, a magnetic field strength of approximately 0.14 A/m was transmitted 32 cm above the bed [27].

Resonant inductive coupling is a type of inductive coupling that is set by tuning the resonance frequency of transmitting and receiving antenna to a predetermined range. Whereas the distance between the transmitting and receiving antenna for conventional inductive coupling without resonance matching must be very close for efficient power transfer, resonant inductive coupling transfers magnetic field flux from the transmitting antenna to the receiving antenna by resonance matching, resulting in a relatively long distance of transfer.

The theoretical efficiency of resonant inductive coupling is 40−60% in several meters apart [30]. Zhang et al. fabricated a loop-shaped transmitting and receiving antenna tuned to 13.56 MHz and wirelessly operated light-emitting diodes and photodiodes for implantable tissue oximetry sensors connected to a microcontroller. The wireless power circuit diagram is presented in Figure 2g [15]. Gutruf et al. used the same wireless circuit to drive the implantable optogenetic device. As shown in Figure 2h, an input power of 8 W was applied to receive a voltage of 2.6 V at 6 cm from the transmitting antenna [28].

Power transfer by non-resonant type inductive coupling provides advantages for simple components and has high efficiency over short distances, but is less suitable for applications requiring power transfer over long distances; i.e., greater than tens of centimeters [21]. Also, precise alignment between transmitting and receiving antenna is needed in order to share the magnetic field to achieve high efficiency [31]. The resonant inductive coupling has advantages in longer transfer lengths. However, it is necessary to precisely design the antenna for tuning resonance frequency and complex implementations may be required [30].

#### 2.1.2. Energy Harvesting

Energy harvesting technologies that directly produce power inside the device system are continuously being developed for the implementation of sustainable wireless wearable sensors, thereby eliminating the need for an external power source [32,33]. There are two ways to implement energy harvesting in a wireless sensor system. One way is to use energy harvesting devices as a power supply of connected sensors [4,34,35,36,37]; the other way is to use energy harvesting devices as a self-powered sensor that measures the voltage or current generated by external stimuli as a sensing signal [38,39,40]. By introducing energy harvesters as the power supply of sensors, the sustainability problems of sensor nodes in wireless sensor networks can be achieved. Srbinovska et al. used photovoltaic cells as a power supply for operating temperature and humidity sensors [36]. Leonov et al. demonstrated a wearable electroencephalography (EEG) sensor operated by the power harvested by the thermoelectric generator attached to the skin [37]. Recent research focused on using energy harvesters (especially nanogenerators) for self-powered sensor systems. For example, Figure 2i shows a triboelectric nanogenerator that functions as a pressure sensor that measures generated currents. As a power source, such energy harvesters can generate high momentary power about 365 V and 65 µA, but it is too high and momentary value for the sensor operation, thus these energy harvesters are more appropriate for the self-powered sensors [29]. More details of self-powered sensor systems will be discussed in the section on applications of wireless sensors.

### 2.2. Wireless Data Communication

Interest is increasing in wireless data transfer for checking data obtained through wearable sensors with computers or smartphones in real-time and to efficiently deliver the vast amount of data stored by sensor systems to the outside world [41]. Various methods for the wireless transfer of data from sensors that detect human signals to a medium that can receive the data are being studied. Appropriate methods are used for the sensor system, depending on the distance between the sensor and the signal receiver, the medium, bidirectionality, and multiple connections (Table 1).

#### 2.2.1. Radiofrequency Identification (RFID)

RFID, also called an electronic tag, is a method that identifies information using radiofrequency. RFID technology refers to technology that uses radio waves to recognize short-range or contactless information. RFID system requires a tag and a reader [60,67]. The tag consists of transceivers and chips, or an antenna can be used as a “chipless RFID” without chips. The RFID can be classified into active, passive, and semi-active tags according to function and power availability [68]. This information is used to identify tagged targets. The tags can be read at a distance of 15 m or less and the range changes according to the frequency. RFID, which reads and communicates chip information only with the power of a reader, is called passive RFID. Recently, passive RIFD has been widely used as a data transmission method for wearable wireless sensors [18,42,43,44,45,46,47]. Depending on the frequency band, RFID can be divided into Low-Frequency Identification (LFID), High-Frequency Identification (HFID), and Ultrahigh-Frequency Identification (UHFID) that use frequencies from 120 to 140 kHz, 13.56 MHz, and 868 to 956 MHz, respectively [68,69]. Since only a radiofrequency antenna can be implemented as a component, an integrated wireless sensor system can be easily realized through the design of the antenna. However, one shortcoming is that the range of a signal is short and complicated signal processing is impossible. Nappi et al. introduced a flexible sensor that can detect the pH of human skin that can be attached to the skin for wireless monitoring. By integrating the pH sensor and an RFID antenna, a low-cost battery-free device was fabricated that can operate in the UHF band (868−956 MHz). This device can transmit pH data collected wirelessly at a distance of up to 1 m [70].

#### 2.2.2. Bluetooth

Bluetooth operates at frequencies between 2.4 GHz and 5 GHz and uses electromagnetic waves for data transmission. In addition to low power and cost, the Bluetooth has the advantage of having a wide transmission range and fast speed, with a maximum transmission distance of 100 m and a maximum transfer rate of 24 Mb/s [68,69]. Furthermore, since microcontroller modules, including Bluetooth communication, are commercially available in various sizes, they can be easily integrated into existing sensors and thus occupy a large part of the wearable wireless sensor market [5,48,49,50,61,71]. Tian et al. integrated the sensor and Bluetooth module into a battery-free wireless energy harvesting device and attached it to metamaterial textiles. With the power received from the device, the Bluetooth-based sensor could wirelessly transmit temperature and humidity values to a smartphone [72]. However, Bluetooth is difficult to miniaturize, since it is deployed in the form of a circuit board that includes a microcontroller unit. Along with the miniaturization of the device, low power consumption is also an important consideration in wearable sensor devices. The sensor device using the classic Bluetooth module consumed higher power than other wireless communication methods such as Zigbee. Devices using Bluetooth Low Energy (BLE) modules that consume low power have recently been used to solve the power consumption problem [51,52]. Gargiulo et al. used a low-power Bluetooth module for wireless connectivity and was designed to be suitable for long-term monitoring during daily activities. Also, this device consumed 30 mW during operation [52].

#### 2.2.3. Near-Field Communication (NFC)

NFC is contactless near-field wireless communication that has a working distance of approximately 20 cm that facilitates the simultaneous transmission of power and data. The advantages of NFC include its compatibility with mobile electronic devices and its high level of security due to its short transmission distance. NFC requires very low power and can operate without batteries in passive mode. NFC can also communicate with active devices, such as smartphones, to read/write NFC tags with components such as chips, antennas, and sensors [41]. Because of these advantages, wearable NFC devices are widely used for wireless transmission [53,54,55,56]. However, the passive type of NFC, which has a short transmission distance and is operational only when power is applied from an external source, can be a disadvantage, depending on the application of the sensor. Xu et al. fabricated patches to measure the concentrations of calcium and chloride ions in various biofluids, such as urine, tears, and sweat. When the NFC-enabled smartphone is brought closer to an NFC antenna, it supplies power wirelessly and transmits the measured data back to the smartphone through the NFC antenna as a battery-free system. Typical NFC-enabled sensors require approximately 200 µW during operation and 3 µW during standby state, so the smartphones can be used as a power supply and data reader [73].

#### 2.2.4. Zigbee

Zigbee is wireless technology designed for networks in the personal area. The Zigbee device uses a mesh network method, which enables communication through multiple intermediate nodes over a wide distance (~100 m) using low power [68]. Wireless devices using Zigbee operate in radio bands of 868 MHz, 915 MHz, and 2.4 GHz. Zigbee has the advantage of being relatively simpler and cheaper than WPAN technologies, such as Bluetooth and Wi-Fi network. Zigbee has a relatively slow data rate of 250 kb/s and can reach up to 20−30 m indoors. Several wireless wearable sensor devices use Zigbee for data transmission [57,58]. Llorente-Alonso et al. developed a sensor that can measure the acidity of the environment whose color changes according to the pH of the surrounding environment. At this time, the signal converted from the optical signal to the current signal through the photodiode is transmitted to the portable device using Zigbee wireless communication [57]. Lee et al. used low-power Zigbee modules, IEEE 802.15.4. This module is quite small and it consumes about 19 mA in receive mode and 17 mA in transmit mode to monitor electrocardiogram (ECG) using conductive fabrics and active electrodes [59]. However, Zigbee technology has some challenges that it is only suitable at low data rates and short coverage.

#### 2.2.5. Resonant Antenna

The resonant antenna is a radiative component in RLC circuits, which consist of a resistor (R), inductor (L), and a capacitor (C). Since the radiative properties of a resonant antenna are dependent to the capacitance and resistance of the RLC circuit, the change in these parameters by physical or chemical factors affect the change in radiative properties of resonant antenna, such as the resonant frequency, resonance bandwidth, or reflection intensity [41]. The resonant antennas have the advantage of manufacturing antennas in planar structures without the need for separate bulky device chips and energy storage. Thus, the resonant antennas are easy to miniaturize, unlike other communication systems. Wireless devices can also be easily implemented by replacing materials in antenna electrodes or modifying designs. For these reasons, many groups are conducting extensive research on wearable wireless sensors based on resonant antennas [60,62,63]. Demonstrations of resonant antenna as a data communication method are also presented in the next sections.

#### 2.2.6. Optical Communication

Optical communication enables wireless communication using light in the infrared, visible and ultraviolet ranges. In general, RF technology is most widely used in the field of wireless communication. The RF band is strictly regulated by the electromagnetic spectrum range between 30 kHz and 300 GHz. As the demand increases for wireless applications and services with large amounts of data, demand for RF spectrum exceeds supply leading to spectrum congestion. The optical data communication can be an alternative to alleviate RF communication’s problem. Compared to RF communication, optical communication is available in a wide bandwidth frequency and has a high spatial resolution and the robustness to electromagnetic interference. Recently, several groups reported wireless wearable sensor devices using optical communication [15,64,65,66]. Rachim et al. Introduced a device for wireless monitoring of electroencephalogram (EEG) using visible light communication. The device uses radiation-free communication modules to enhance the stability of EEG devices that require long-term monitoring. Also, using a 30 Hz smartphone camera, it showed EEG data transmission at a transmission speed of 2.4 kbps at a maximum distance of 4 m [64].

## 3. Recent Developments of Wireless Sensors

In this section, various types of sensors including physical, chemical, and electrophysiological sensors are discussed, from their sensing mechanisms to the use of wireless functions for power supply and data communication.

### 3.1. Physical Sensors

Physical sensors can detect and monitor the surrounding environment and apply physical information (e.g., pressure, temperature, and strain) to various fields, such as wearable electronics, soft robotics, Electronic skin, and real-time health monitoring [74]. Recently, wearable sensor electronics have been developed that are stretchable and flexible, since a sensor needs to be able to conformally deform as the body deforms during physical activity. Also, to minimize discomfort, movement should be able to drive wearable sensor devices wirelessly. Most of these physical sensors sense temperature, pressure, and strain.

#### 3.1.1. Temperature Sensors

Body temperature is an important parameter that is associated with various diseases. Examples of diseases that can be diagnosed by body temperature include heat stroke, congestive heart failure, malignant tumor, fever, hyperthermia, and infection [75]. Therefore, accurate real-time monitoring of body temperature can help to diagnose sudden illnesses at an early stage, such as heart attacks [41]. Therefore, the device needs to have fast response speed, high sensitivity, a wide detection range, and wearable characteristics. In addition, the device needs to be flexible, stretchable, and wireless for maximum comfort of the user. In consideration of these characteristics, wearable temperature sensors have recently used various nanomaterials as heat sensing elements [76,77]. In addition, sensors can be divided into three categories according to the method of temperature detection: pyroelectric temperature detector, resistive temperature detectors, and thermistor. In pyroelectric temperature detectors, the polarization of a material caused by changes in temperature creates an electric field, which is called the pyroelectric effect [78,79,80,81]. Among all temperature sensors, resistive temperature detectors are the most commonly used. The principle of resistive temperature detectors is that the electrical resistance of metal changes with temperature [82,83]. Unlike a resistive temperature detector, a thermistor has high temperature resolution because the resistance changes nonlinearly with temperature [84,85,86]. There are cost advantages to the thermistor, but its temperature range is limited [41].

**Figure 3:**
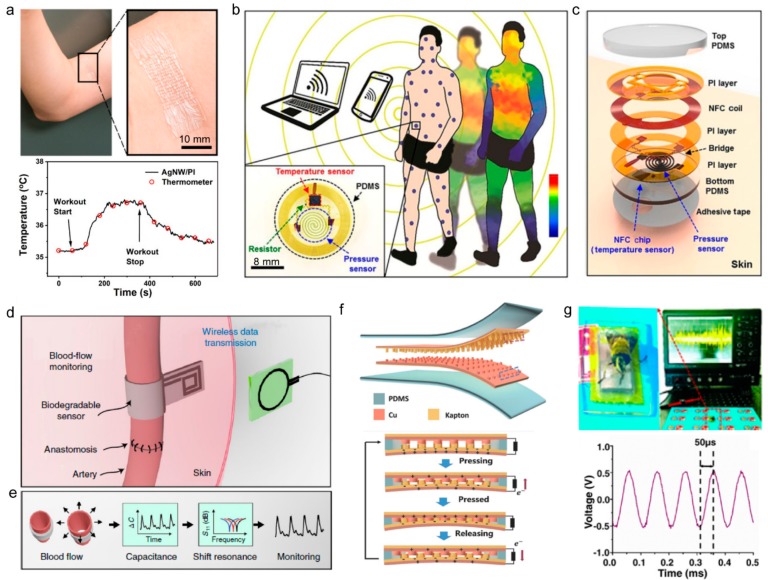
Physical sensors with wireless functions. (**a**) Top: photograph of AgNW/PI temperature sensor is attached to the skin near biceps. Bottom: temperature recorded by the temperature sensor and IR thermometer during biceps workout (Reproduced with permission [87]. Copyright 2019, American Chemical Society). (**b**) Illustration of a collection of thin, conformable skin-mounted sensors distributed across the body, with continuous, wireless transmission of temperature and pressure data in a time-multiplexed fashion. Inset: Top-view photograph of a representative sensor (Reproduced with permission [27]. Copyright 2018, The American Association for the Advancement of Science). (**c**) Exploded view schematic illustration of the device structure (Reproduced with permission [27]. Copyright 2018, The American Association for the Advancement of Science). (**d**) Post-operative monitoring of arterial pulsations after surgery (Reproduced with permission [44]. Copyright 2019, Springer Nature). (**e**) Sensing concept. The arterial pulsation results in a change in vessel diameter that is measured by the capacitive pulse sensor mounted around the artery. The change in capacitance results in a shift of the resonance frequency of the RLC circuit. This shift is measured wirelessly through the skin using an external reader coil (Reproduced with permission [44]. Copyright 2019, Springer Nature). (**f**) Schematic diagram of triboelectric nanogenerator (top). Schematic diagram of the working principle of self-powered ultrasensitive pulse sensor (bottom) (Reproduced with permission [88]. Copyright 2017, John Wiley and Sons). (**g**) Top: The optical image of bee wings on the pulse sensor, with the output performance driven by the bee wings (frequency, ≈ 200 Hz) display on the oscilloscope in real time. Bottom: The output voltage of the pulse sensor with the extremely high frequency of 10 kHz (Reproduced with permission [88]. Copyright 2017, John Wiley and Sons).

Cui et al. developed a flexible, breathable thermal resistant temperature sensor using silver nanowires. This sensor was patterned in a kirigami structure and can withstand large tensile strain (up to 100%). This temperature sensor measures changes in resistance as the temperature changes and is attached to the skin to monitor muscle temperature during exercise, as shown in Figure 3a [87]. Han et al. developed a flexible adhesive sensor that measures skin temperature and pressure in real-time. Patients who need to stay in bed for long periods of time are more likely to develop pressure ulcers or bedsores. This device is designed to adhere thinly to the skin for continuous wireless monitoring of temperature and pressure (Figure 3b) [27]. The pressure sensor has a spiral structure composed of a silicon membrane that has piezoresistive properties. The temperature sensor uses a resistance thermometer detector integrated into the NFC chip (Figure 3c). Power is supplied by radio frequency (RF) over external antennas and NFC is used to transmit data.

#### 3.1.2. Tactile Pressure Sensors

The pressure in the body can range from weak pressure, such as intraocular pressure or cranial pressure, to strong pressure, such as weight pressure on the foot [89,90]. Strain also includes large range physical activities, such as stretching, and small range activities, such as vibration or pulsing of the vocal cords. By measuring the pressure and strain of the human body, various diseases, such as cardiovascular disease, eye disease, heart failure, and muscle damage breathing problems, can be monitored. Accordingly, along with the development of highly sensitive wearable pressure and strain sensors for healthcare purposes, research is being carried out to make these devices flexible, stretchable, and wireless [91,92]. Pressure sensors and strain sensors can be classified into piezoresistive, capacitance, and piezoelectric methods. Piezoresistive is the principle that the resistance of material changes when pressure is applied, and the resistance varies depending on the geometrical structure, tunneling resistance, and contact resistance that responds to the applied pressure [93,94]. Advantages of piezoresistive include simple technologies and equipment, easy read-out signals, and a wide sensing range. The capacitance-based pressure sensor uses capacitors and capacitance, depending on the thickness of the dielectric that grows as pressure increases and thickness decreases [17,95]. The capacitance-based pressure sensor has the advantage of having high sensitivity that can detect static pressure and low hysteresis. The importance of wireless data transmission of wearable devices in healthcare is growing. Recently, sensors that transmit changes in the resonant frequency of antennas through wireless communication by external physical stimuli without a power source have been developed [18,42,43,44,45]. Kou et al. developed a poly (dimethyl-siloxane) (PDMS)/graphene dielectric layer and inserted it between the antenna electrodes patterned with Cu. Then, as the thickness of the dielectric layer changes due to pressure, so does the resonant frequency of the antenna. Kou et al. developed a sensor that measures pressure by sensing the alteration in pressure The sensitivity of this sensor is 2.2 MHz/kPa and has an operating range of 0–500 kPa [42]. Boutry et al. fabricated a pressure sensor by inserting a dielectric fabricated from pressure-sensitive micro-structured poly (glycerol sebacate) between split-ring resonator-type antennas. This pressure sensor can be used to monitor blood pressure in the postoperative arteries (Figure 3d). Information from the passive pressure sensor was read through a reader coil connected to the network analyzer, as shown in Figure 3e [44]. Additionally, self-powered physical sensors that use nanogenerators as sensors are being developed. In general, triboelectric nanogenerators (TENGs) generate static electricity from friction and electrostatic induction between two types of surface materials. Because TENG creates voltage only when there is friction resulting from physical strain, this voltage can be used as a signal of deformation. Han et al. developed self-powered pulse sensors for cardiovascular disease (Figure 3f). Ranging from the vibrational frequency of bee wings (approximately 200 Hz) to the high mechanical frequency generated by a speaker (10 kHz), the pulse sensors can accurately detect and confirm the pressure as an electrical signal in real-time (Figure 3g). Using Bluetooth chips, communications can wirelessly transmit a pulse waveform to a smartphone or computer and monitor the pulse data in real-time [88]. Thus, the technology that is used to wirelessly transmit information measured by sensors is critical.

#### 3.1.3. Optical Sensors

Optical sensors can detect various characteristics of light, such as frequency, intensity, wavelength, or polarization, and can convert these wide ranges of optical signals into electrical signals [96,97,98]. The optical sensor is not affected by electromagnetic radiation and enables non-invasive diagnosis at relatively large penetration depths. Optical sensors also have the advantage of using low cost, waterproof, and corrosion-resistant elements [99]. The performance of the optical sensor can be evaluated by the sensor’s sensitivity, response time, and selectivity [100]. One of the most famous optical sensors in terms of diagnosis of health is a photoplethysmography (PPG) sensor that can measure blood oxygen saturation, pulse rate and blood pressure based on a photodetector. The PPG sensors typically consist of a photodetector and two light-emitting diodes (LEDs) with different emission wavelengths. The principle of the PPG sensor is to measure the oxygen saturation of arteries using the difference in absorbance between oxy-hemoglobin (HbO_2_) and deoxy-hemoglobin (Hb) in the blood [101,102]. For example, infrared light has a higher absorbance at oxy-hemoglobin (HbO_2_) and red light has a higher absorbance at deoxy-hemoglobin (Hb). The frequency of the fluctuating light absorption (i.e., the AC component of the PPG signal) represents the pulse rate, and the magnitude of the AC component of the PPG signal corresponds to the blood pressure caused by contraction and relaxation of the heart. Recently, various wireless wearable devices have been developed for health monitoring using PPG sensors [65,103,104]. Azhari et al. developed a patch-type wireless wearable pulse oximeter. The patch incorporates a PPG sensor consisting of red light with a wavelength of 625 nm, infrared light at 865 nm, and a photodetector. This patch-type device can be worn on the forehead to measure pulse rate and blood oxygen saturation. Also, the digitized data was wirelessly transmitted to the external computer via Bluetooth module, enabling real-time health monitoring [103]. Near-infrared spectroscopy (NIRS) sensors are also one of the representative optical sensors that provide high spatial resolution. In general, the method of measuring the NIRS signal in the NIRS sensor is similar to the PPG sensor. Human tissue is relatively transparent in the near-infrared region between 650−1000 nm [105], of which oxygen saturation is measured using the difference in the main absorbers’ absorbance, Blood chromophores of Oxy-hemoglobin (HbO_2_) and deoxy-hemoglobin (Hb) [106]. As such, using near-infrared (NIR) light with low attenuation due to transparency, it has the advantage of increasing the penetration depth of light, and sending/receiving signals from larger arteries. However, NIRS sensors have the disadvantages of being difficult to miniaturize and expensive compared to PPG sensor devices [107,108]. Guo et al. reported a wireless sensor system capable of simultaneously recording surface electromyography (sEMG) and NIRS signals. The device consists largely of three parts: the part that amplifies the measured signal, the part that digitizes the measured signal, and the part that transmits digitalized signal wirelessly. Also, sEMG-NIRS integrated device was attached to a human’s arm to transmit the digital signals according to the movement of the arm muscle to the PC wirelessly using the Bluetooth communication [109].

### 3.2. Chemical Sensors

Wearable sensors have received a significant amount of attention due to their potential applications in health and environmental monitoring. Wearable sensors can accurately detect various chemical signals in the human body that are associated with disease biomarkers as well as environmental pollutants and toxic gases in a continuous manner [41]. Until now, different chemical sensors have been used in healthcare for monitoring biochemicals in blood, sweat, tears, urine, and saliva with the extraction of these biofluids. These conventional chemical sensors are associated with discomfort and sometimes cause pain to the patient. In addition, it is difficult to continuously sense various diseases and limit the daily activities of patients who must visit the hospital. Improvements to wireless chemical sensors, such as minimizing patient discomfort and real-time monitoring, are needed.

#### 3.2.1. Amperometric Sensors

**Figure 4:**
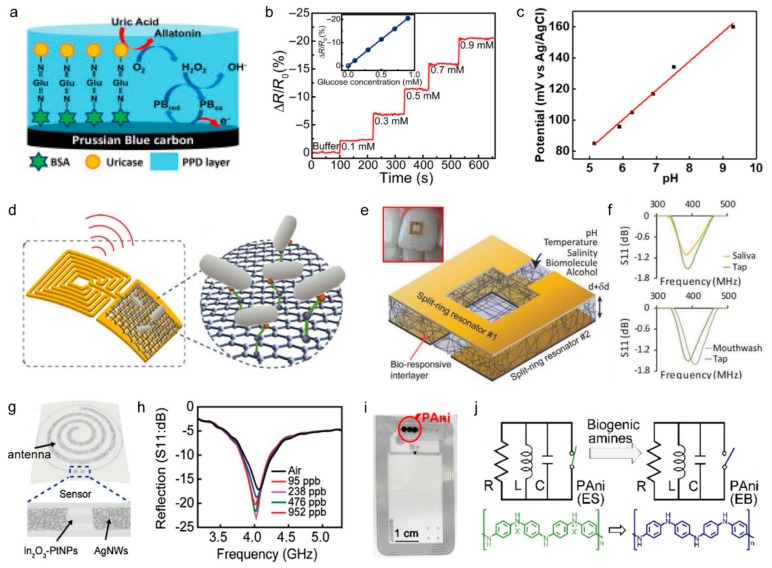
Chemical sensors with wireless functions. (**a**) Reagent layer of the chemically modified printed Prussian-Blue carbon working electrode containing uricase for SUA biosensor (Reproduced with permission [112]. Copyright 2015, Elsevier). (**b**) Real-time continuous monitoring according to the glucose concentrations (inset, calibration curves of the glucose sensor) (Reproduced under the terms of the CC BY-NC license [3]. Copyright 2018, the authors, The American Association for the Advancement of Science). (**c**) Potentiometric performance of the pH sensor (Reproduced with permission [115]. Copyright 2018, Elsevier). (**d**) schematic of the sensing element and wireless readout. A magnified illustration is binding of pathogenic bacteria by peptides self-assembled on the graphene nanotransducer (Reproduced with permission [116]. Copyright 2012, Springer Nature). (**e**) Schematic of broad-side coupled split-ring resonators with an interlayer of silk film or responsive hydrogel. Interlayers swell and absorb surrounding solvent (changing thickness and dielectric constant) and result in a change in resonant frequency and amplitude of the sensor. Inset: Trilayer sensor adhered to a human subject’s tooth for in vivo monitoring of ingested fluids (Reproduced with permission [18]. Copyright 2018, John Wiley and Sons). (**f**) Thin interlayer (≈ 1.2 µm) response on Subject 1 to various liquids. Changes to frequency and magnitude are seen in each case (Reproduced with permission [18]. Copyright 2018, John Wiley and Sons). (**g**) Schematic illustration of the transparent, flexible alcohol sensor integrated with a wireless antenna (Reproduced with permission [71]. Copyright 2018, Elsevier). (**h**) The experimental results (reflection value, S11) of the wireless sensor before and after exposure to the ethanol vapor of 95, 238, 476, 952 ppb (Reproduced with permission [71]. Copyright 2018, Elsevier). (**i**) Photograph of an NFC tag modified with printed PTS-PAni (Reproduced with permission [117]. Copyright 2018, American Chemical Society). (**j**) The circuit of the modified NFC tag. The amine gas released from spoiled meat dedopes PTS-PAni and increases the resistance of the device and thus switches the readability of the NFC tag (Reproduced with permission [117]. Copyright 2018, American Chemical Society).

Traditional chemical sensors are divided into amperometric and potentiometric sensors according to the method of signal measurement. Amperometric sensing measures the change in current caused by analyte interaction with sensors while a constant voltage is applied to the sensor. This type of sensor uses functionalized enzymes or antibodies on the working electrodes that can induce a charge-interaction with specific molecules to sense biomolecules such as glucose, proteins, alcohols, lactate, or gas molecules [43,46,110]. Chemiresistive sensors are a type of amperometric sensor in which an active channel exists between two conductive pads, and senses current change during interaction with the molecule. Amperometric sensors are being actively researched as wearable sensors that are attached to body parts, including skin, eyes, and teeth, which can continuously interact with secreted body fluids that contain various biomolecules. For a stable supply of constant voltage and data reception, amperometric sensors typically have been integrated with a Bluetooth-enabled microcontroller [111,112]. Kim et al. fabricated a uric acid sensor by coating a uricase on the surface of the working electrode (Figure 4a) and then connecting the sensor to a Bluetooth-enabled microcontroller powered by a battery, and the device was attached to a mouthguard in order to detect uric acid in saliva. The resulting wireless sensor could detect uric acid at a concentration of 50 µM, and the signal was transmitted to a computer via a Bluetooth module for four hours [112]. In order to achieve miniaturization and to ensure stability in a wet biofluidic environment, a significant amount of research was previously conducted in which power was supplied by wireless power transfer without an internal battery [3,26,27,113,114]. Park et al. developed a sensor system using the amperometric glucose sensing (Figure 4b) and presented the signal by the operation of a light-emitting diode that was wirelessly powered by the inductive coupling of an antenna [3].

#### 3.2.2. Potentiometric Sensors

Potentiometric sensing measures the potential change between the working and reference electrodes along with the electrolyte. Potentiometric sensing is often used to measure ion concentrations in the body [118]. Dang used graphite as a working electrode and Ag/AgCl as a reference electrode to measure pH (Figure 4c). The sensor was driven by wireless power transfer and had a sensitivity of approximately 11 mV/pH; this potentiometric signal was output to a smartphone [115]. Lee et al. used a Na+ selective membrane as a working electrode and Ag/AgCl as a reference electrode to detect sodium intake. The sodium intake sensor system transfers signals to a smartphone using a Bluetooth-enabled microcontroller with a sensitivity of 188 mV/decade [119].

#### 3.2.3. Radiofrequency-Based Sensors

As wireless technologies have been applied to wearable chemical sensors, research has also been conducted to design an antenna’s RLC circuit in which the resonance frequency of the antenna changes with analyte concentration [18,116,117,120,121]. Mannoor et al. designed some part of receiving antenna with bacteria binder so that the resonance frequency changed according to bacterial concentration (Figure 4d). This bacterial sensor was attached to tooth enamel in order to wirelessly detect bacteria attached to the teeth [116]. Tseng et al. fabricated a dielectric layer of the split-ring resonator type antenna that varies in thickness, depending on biomolecules or ions (Figure 4e); consequently, this resulted in the production of a sensor that detects changes in resonance frequency and reflection values of the split-ring resonator. Figure 4f shows different resonance responses to various liquids [18]. Kim et al. fabricated a flexible alcohol sensor with chemiresistive electrodes made of platinum nanoparticle-decorated indium oxide thin film and used this sensor as a part of an antenna coil, as shown in Figure 4g. The reflection value of antenna increased, and the resonance frequency slightly decreased with increasing glucose concentration, from 95 to 952 ppb (Figure 4h). This device system was able to detect glucose in tears at a concentration of 1 µM [71]. Ma et al. developed a sensor that can monitor food spoilage by transmitting a signal to a smartphone from an NFC antenna in a food box. As shown in Figure 4i, an antenna, partially composed of conductive polymer polyaniline doped with p-toluene sulfonic acid (PTS-PAni), and PTS-PAni acted as a variable resistor that changes resistance according to the concentration of volatile biogenic nitrogen, which consequently changes the resonance frequency of the antenna (Figure 4j) [117].

### 3.3. Electrophysiological Sensors

Electrophysiological signals generated in the body provide essential information that can be used to assess organ functionality or to diagnose disease [122]. For example, electroretinography (ERG) allows for the diagnosis of visual functions by monitoring how electrical activity generated in photoreceptor cells responds to light stimuli [123]. Electromyography (EMG) and electro-cardiography (ECG) can be used to monitor muscle disorders in Parkinson’s patients or for diagnosing heart arrhythmia [124,125]. In addition, as research has been recently performed on brain-machine interfaces and neural networks, electrophysiological sensing of the brain can be used to better understand the brain [126,127,128]. Therefore, the development of wearable forms of electrophysiological sensors capable of obtaining high signal-to-noise ratio (SNR) results and continuous data by conformal contact to the surface or interior of organs, including skin, has been studied.

**Figure 5:**
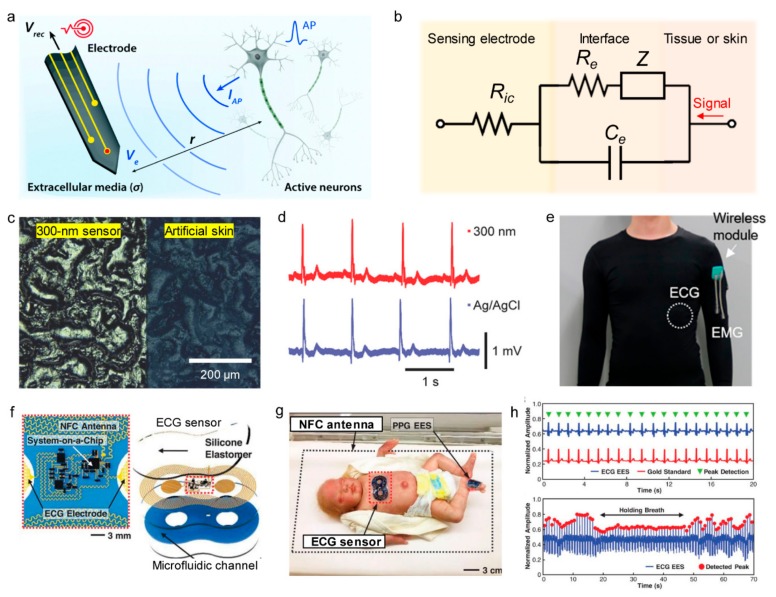
Electrophysiological sensors with wireless functions. (**a**) Schematic overview of bioelectronic recording. Upon firing of APs, electrically active neurons inject charges into extracellular media with the corresponding extracellular potential and local field potential. The resultant potential V_e_ within electrolytic tissue media applied on the electrolyte-electrode interface is transmitted via electronic interconnects and recorded as the output signal, V_rec_ (Reproduced under the terms of CC BY-NC license [129]. Copyrights 2019, Royal Society of Chemistry). (**b**) Equivalent circuit diagram of the interface (Randles circuit) between the sensing electrodes and the biological system. (**c**) Optical image of 300 nm dry, thin-film electrode laminated on artificial skin. The right side of the image shows the bare artificial skin, while the left side shows the laminated sensor (Reproduced with permission [130]. Copyright 2018, John Wiley and Sons). (**d**) A series of ECG spikes measured with 300 nm thin film (top, red), and wet adhesive Ag/AgCl gel (bottom, blue) sensors (Reproduced with permission [130]. Copyright 2018, John Wiley and Sons). (**e**) Multimodal sensing suit composed of EMG and ECG sensors with wireless transmission module for long-term continuous monitoring of physiological activities (Reproduced with permission [131]. Copyright 2019, American Chemical Society). (**f**) Schematic illustration of wireless, battery-free modules for recording ECG data. The ionic liquid in the microfluidic channel contains blue dye for visualization purposes (Reproduced under the terms of CC BY license [132]. Copyright 2019, the authors, American Association for the Advancement of Science). (**g**) neonatal intensive care unit setting with a binodal (chest and foot) deployment of skin-like wireless devices designed to provide the same functionality and measurement fidelity (Reproduced under the terms of CC BY license [132]. Copyright 2019, the authors, American Association for the Advancement of Science). (**h**) Top: ECG signals acquired simultaneously from an ECG sensor (blue) and a gold standard (red), with detected peaks (green). Bottom: Respiration rate extracted from oscillations of the amplitudes of peaks extracted from the ECG waveforms (Reproduced under the terms of CC BY license [132]. Copyright 2019, the authors, American Association for the Advancement of Science).

Figure 5a shows the interface between tissue and electrodes and its signal transduction during electrophysiological sensing. As opposed to electronic devices in which the electrons act as charge carriers, biological tissues have electrolytic media in which electrophysiological signals are mainly transmitted by ionic flux. Therefore, for electronic devices to accept signals from biological tissues, an interface is required in which ionically carried signals can be exchanged with electronically-carried signals. The equivalent circuit of a tissue-electronics interface is shown in Figure 5b. The potential *V_e_* generated by the ionic flux in the cell across the surrounding electrolyte is expressed as [129]:

$$V_{e}=\frac{I_{AP}}{4\pi \sigma r}$$

in which *I_AP_* is the transmembrane current induced by an action potential, *r* is the distance between the target cell and the electrode surface, and *σ* is the conductivity of the electrolytic media. In this case, the recording potential *V_rec_* in the recording electrodes is expressed as:

$$V_{rec}=\frac{I_{AP}}{4\pi \sigma r}\left[\left(\frac{1}{R_{e}}+sC_{e}\right)R_{ic}\right]$$

in which *s* is the frequency of action potentials, *C_e_* is the capacitance of the tissue-electronics interface, and *R_ic_* is the resistance of the interconnection electrodes. To increase the quality of the recording signals, use of low resistance and high capacitance recording electrodes is required and the recording electrode must be placed close to the cell. Also, achieving a low impedance between the recording electrode and the electrolytic media is one of the most important goals. Since impedance is inversely proportional to the contact area, studies have been performed to make the electrodes conform to body parts by fabricating stretchable, thin electrodes for physiological sensing. Nawrocki et al. fabricated a very thin, Au thin film sandwiched between the Parylene-C polymer double layer to the 300 nm level, and used the thin film as an electrode for ECG and EMG sensing. Previously, gel-type electrodes were used for conformal contact, but the developed electrode was very thin, which reduced stiffness and allowed conformal contact according to the microscopic skin tissue, as shown in Figure 5c. The conformal contact of electrodes resulted in a lower impedance value of 23.3 kΩ at 1 kHz and an SNR value of 21.09, which is an improvement over the Ag/AgCl gel electrodes. As shown in Figure 5d, there is no significant difference between the two ECG signals recorded by thin-film electrodes and the Ag/AgCl electrodes [130]. Subsequently, research was conducted that integrated wearable electrophysiological sensors with wireless technologies. Jin et al. applied a conductive gel on the surface of a composite where silver flakes and PVDF nanofibers were dispersed inside the elastomer and used it as a flexible bio-interfacing electrode for electrophysiological recording (Figure 5e) [131]. Chung et al. used flexible metal mesh microstructures with fractal geometry as recording electrodes in skin-interfacing ECG sensors. The antenna was fabricated to be stretchable and to receive power from external sources with resonant inductive coupling and to be able to charge NFC chips in a battery-free fashion (Figure 5f). The sensor is attached to an infant for continuous neonatal care, and the NFC antenna is installed under the baby incubator, so the sensor can be powered anywhere inside the incubator. 

The data signal detected by the incubator is transferred to a computer through Bluetooth communication (Figure 5g). This type of sensor allows for the heartbeat and respiration of an infant to be monitored in real-time (Figure 5h) [132].

Unlike electrophysiological sensing at the organ or muscle level, neuron-level electrophysiological sensing requires reading the signal from a single neuron independently; therefore, it is essential to reduce the size of the recording electrode (increasing spatial resolution). In this case, the impedance of the electrode increases; thus, specific electrode materials, such as iridium oxide [133], platinum [134], poly(3,4-ethylenedioxythiophene)-poly(styrene sulfonate) (PEDOT:PSS) or PEDOT [135], which have low areal impedance and biocompatibility or a surface modification such as platinum black [136] that can increase the surface area have been used.

## 4. Applications of Wireless Sensors

In this section, numerous studies of wearable devices in which wireless sensor systems are being actively applied are discussed. Wireless technologies for each applied body part or target analytes of wearable devices are investigated, and the progress of each application is discussed. The reviewed works in each application are summarized in Table 2.

### 4.1. Electronic Skins

**Figure 6:**
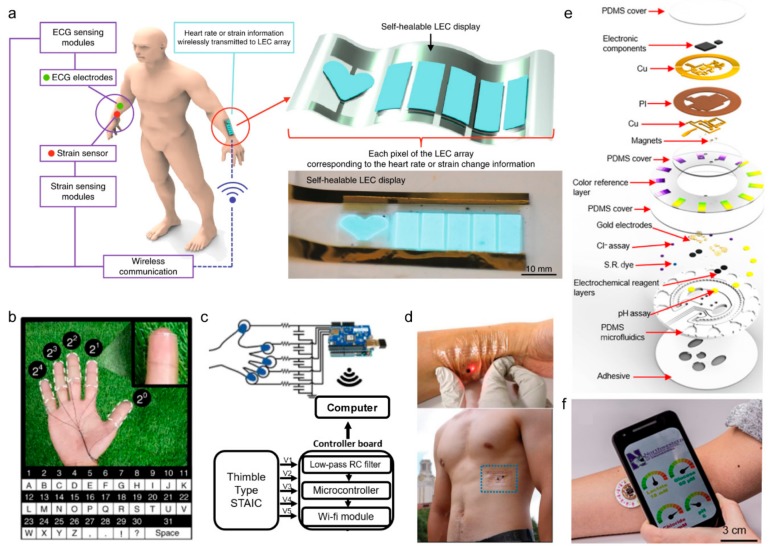
Electronic skins. (**a**) Overview of the system with sensors wirelessly communicating values to the display (Reproduced with permission [137]. Copyright 2018, Springer Nature). (**b**) Self-cleanable, transparent, and attachable ionic communicators were attached on the fingers and connected with wires to a controller board. A different order of a binary system was assigned to each finger, and the letters were pre-coded in the microcontroller (Reproduced with permission [138] Copyright 2018, Springer Nature). (**c**) Block diagram of the communicator based on STAICs. STAICs were connected to a controller board which contains an RC low-pass filter, a microcontroller, and a Wi-Fi module (Reproduced with permission [138] Copyright 2018, Springer Nature). (**d**) Top: A photo of the assembled ECG e-tattoo. Photos of a wireless e-tattoo with LED worn on the skin powered by a wireless NFC reader concealed beneath the white paper: compressed. Bottom: A picture of the battery-free ECG e-tattoo applied at the lower rib cage of a male subject (Reproduced with permission [53]. Copyright 2019, John Wiley and Sons). (**e**) Schematic illustrating the exploded view of the complete hybrid battery-free system. PI, polyimide; S.R., sweat rate (Reproduced under the terms of the CC BY-NC license [139]. Copyright 2019, the authors, published by The American Association for the Advancement of Science). (**f**) A phone interface that illustrates wireless communication and image acquisition (Reproduced under the terms of the CC BY-NC license [139]. Copyright 2019, the authors, published by The American Association for the Advancement of Science).

Most of the human body is covered by skin. Accordingly, skin-attachable devices are gaining attention. Skin-attachable electronic devices, also called electronic skin, are a very thin electrode that implements sensors, power supply, and telecommunication components that utilize conventional electronic technology. The advantage of electronic skin is that it is light and easy to bend and stretch. Unlike other wearable electronic devices that have a relatively large volume and weight, an electronic skin is made similar to a thin film and can be evenly attached to curved skin through van der Waals forces [152]. Electronic skin is mainly used to monitor human’s physiological signals by integrating sensors, and wireless technologies are being integrated. Son et al. developed a stretchable electronic skin system that wraps a nanowire conductive network in a self-healable polymer. This device can monitor the strain of skin and is used as an ECG sensor and a light-emitting capacitor (LEC) display array. Physiological data recorded by each sensor were read by the power of a lithium-ion battery and were then wirelessly transmitted to the LEC display array using Bluetooth for real-time monitoring (Figure 6a) [137]. Lee et al. created a transparent, attachable ionic communicator by attaching TENG to each finger (Figure 6b). PDMS and hydrogel were combined chemically to form electrodes that adhere strongly to the skin and that are treated with trichlorosilane (HDFS) to prevent surface contamination. Also, the triboelectricity generated from the contact of TENG electronic skin was transmitted to the external computer through wi-fi as a sensing signal (Figure 6c) [138].

Electronic skin can also be used to detect biomolecules and diagnose disease in the body. The total number of glands in the human body is as high as 100 and sweat plays an important role in the sensing of chemicals in the body. Therefore, it is possible to monitor stress by analyzing sodium in sweat or to diagnose cystic fibrosis by analyzing pH [153]. The analysis of pH can be performed in various ways, including the use of photodiodes and LEDs to measure the color change of pH-sensitive dyes or by the use of capillary force to collect and analyze perspiration on functional materials naturally, and then using LC resonators and the capacitive electrode to measure pH levels wirelessly [63,154]. Also, for athletes who need endurance, it is important to monitor the level of lactic acid in order to maintain high speeds [155]. The enzymatic reaction of lactate oxidase can be used to measure changes in the current [156] or to monitor the level of lactic acid in real-time through the screen printing process [157]. Jeong et al. developed a device that is stretchable and attaches to the skin for electrocardiography, blood oxygen saturation level, heart rate, skin temperature, and hydration (Figure 6d). The reader coil measures the uniaxial strain of the device through the resonant frequency and transmits it to the computer via NFC [53]. Recently, Bandodkar et al. developed a chronometric microfluidic platform that can monitor sweat rate/loss, pH, lactate, glucose, and chloride at the same time; Figure 6e shows the scheme of the device. The platform consists of a disposable soft microfluidic network and a reusable NFC electronic module. It uses a double layer copper-on polyimide substrate and transmits signals to a smartphone using an NFC antenna on a laser-patterned chip with a radiofrequency system (Figure 6f) [139].

### 4.2. Smart Contact Lenses

**Figure 7:**
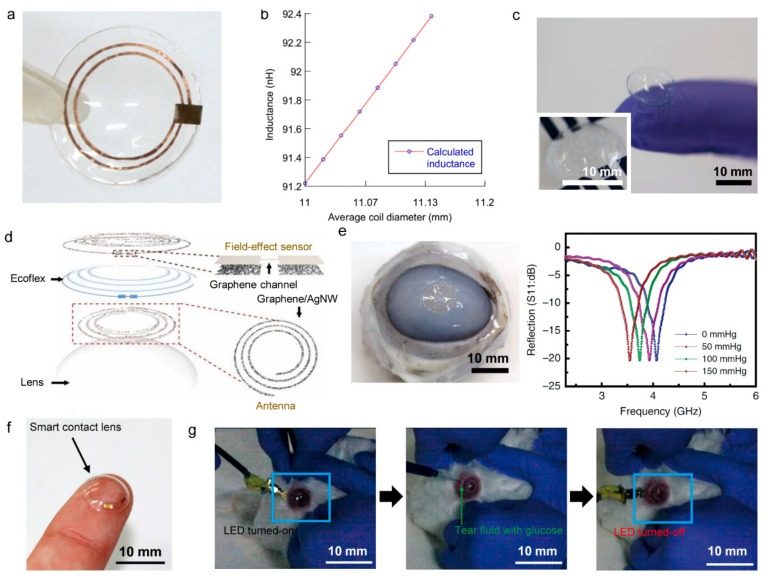
Smart contact lenses. (**a**) The contact lens sensor with sensing elements embedded in a silicone rubber contact lens (Reproduced with permission [140]. Copyright 2014, Elsevier). (**b**) The inductance of the coil as a linear function of the average coil diameter (Reproduced with permission [140]. Copyright 2014, Elsevier). (**c**) A photograph of the contact lens sensor. Scale bar, 1 cm. (Inset: a close-up image of the antenna on the contact lens (Reproduced under the terms of the CC BY license [45]. Copyright 2017, Springer Nature). (**d**) Schematic of the wearable contact lens sensor, integrating the glucose sensor and intraocular pressure sensor (Reproduced under the terms of the CC BY license [45]. Copyright 2017, Springer Nature). (**e**) Photographs of the sensor transferred onto the contact lens worn by a bovine eyeball (Left). Wireless recording of the reflection coefficients at different pressures (Right) (Reproduced under the terms of the CC BY license [45]. Copyright 2017, Springer Nature). (**f**) Photograph of the fabricated soft, smart contact lens (Reproduced under the terms of the CC BY-NC license [3]. Copyright 2018, the authors, American Association for the Advancement of Science). (**g**) Photographs of the in vivo test on a live rabbit using the soft, smart contact lens. Left: Turn-on state of the LED in the soft, smart contact lens mounted on the rabbit’s eye. Middle: Injection of tear fluids with a glucose concentration of 0.9 mM. Right: Turn-off state of the LED after detecting the increased glucose concentration (Reproduced under the terms of the CC BY-NC license [3]. Copyright 2018, the authors, American Association for the Advancement of Science).

Contact lenses are a popular product around the world and make physical contact with the surface of the human eye and tears [158]. Using contact lenses as a wearable device platform, research is currently being conducted for the development of smart contact lenses, which represent wearable electronics with functions such as diagnosing ophthalmic disease and detecting biomolecules in tears. The ultimate goal of smart contact lenses is to continuously monitor the health of the user in real-time through continuous contact with the eyes and tears. Tears contain many biomolecules. Because these biomolecules diffuse directly from the blood, analysis of tears is a good way of non-invasively monitoring human physiological conditions as a substitute for blood analysis [159,160]. Typically, by incorporating chemical sensors into smart contact lenses, the glucose concentration in tears can be measured as a way to diagnose diabetes or cardiovascular disease [11,161,162,163]. However, early studies used a wire to measure glucose concentration. The intraocular pressure sensor is another type of sensor based on smart contact lenses. Intraocular pressure is the most essential factor for diagnosing glaucoma [164]. Therefore, real-time monitoring of intraocular pressure is critical for early diagnosis and treatment of glaucoma [165]. To this end, research has been conducted on the detection of intraocular pressure by integrating a strain sensor into a contact lens to measure the change in force acting on the lens. The most representative methods are strain gauge sensors [166,167] and capacitive measurements [140,168,]. Sensimed produced a contact lens sensor with two strain gauges: a microprocessor and an antenna on a conventional silicon soft contact lens [167]. The method of measuring eye pressure uses a strain gauge to measure the change in the curvature of the cornea due to pressure changes in the eye. The contact lens receives power from the antenna attached around the patient’s eye and transfers the strain gauge information back to the attached antenna. This information is sent to a portable recorder and is then transferred to a computer using Bluetooth. In this way, a total of 86,400 strain gauges can be obtained in 24 h. However, in order to deliver data through the lens, an antenna must be attached around the patient’s eyes (i.e., not truly wireless), which is connected to a bulky recorder for data transmission. Chen et al. combined an inductive Cu coil (L) and a thin film capacitor (C) to form a resonator to wirelessly measure the change in inductance of the coil with an external reader. The soft, deformable LC resonance circuit in the lens detects intraocular pressure by measuring the change in curvature of the cornea. The Cu antenna and resonance circuit are sandwiched between two silicone rubber layer (Figure 7a). The experimental results showed that the response frequency of the contact lens sensor was fast (~8 kHz/mmHg) and changed linearly with changes in intraocular pressure (Figure 7b) [140]. This study demonstrated the potential of wireless electronic devices by demonstrating wireless measurement of intraocular pressure. However, the series of lenses, including the aforementioned contact lenses, are not stretchable because they are based on a thin film of metals and polyethylene terephthalate substrates, which makes them difficult to wear for long periods of time and limits the field of view because components needed for wireless operation, such as antennas, are not transparent. To solve this problem, Kim et al. developed a transparent and flexible smart contact lens capable of wirelessly measuring intraocular pressure and glucose concentration (Figure 7c). Graphene and Ag nanowires were hybridized to form two spiral inductive coils and then silicon elastomers (Ecoflex) were placed between them (Figure 7d). Increased curvature of the cornea due to increased intraocular pressure results in thinning of the dielectric between the spiral coils, which increased the capacitance and inductance, thereby varying the resonant frequency. The signal can be wirelessly received by an external antenna in order to measure a change in resonance frequency according to intraocular pressure. Figure 7e contains the results of an experiment conducted with a bovine eyeball, which shows the change in intraocular pressure through a response measurement of the reproducible resonant frequency [45]. Park et al. reported a smart contact lens that monitors glucose levels through the operation of LEDs. Transparent antennas were fabricated with metal nanowires to ensure visibility and the rigid components required for wireless driving (rectifiers, LED, glucose sensor) were placed and protected on flexible hybrid substrates (Figure 7f). This design provides elasticity to the entire smart contact lens system. By wirelessly transmitting power using inductive coupling, the concentration of glucose can be determined by dimming of the LED light as the resistance of the glucose sensor decreases as the glucose concentration increases. Figure 7g shows a test image of a smart contact lens applied to a rabbit’s eyes. This rabbit showed no abnormal behavior and the lens remained stable, even with eye blinking [3].

### 4.3. Neural Interfaces

The recording of electrical signals generated by neurons or stimulating neurons has significantly evolved over the past decades with the growth of biomedical engineering and micro/nanoelectronics. To understand the brain, one of the main purposes of the neural interface, or brain-computer interface, is to stimulate the nervous system or analyze signals generated in the brain, and to diagnose and treat psychological diseases and various neurological disorders [169]. EEG measures the electrical activity of the brain by touching electrodes on the scalp surface. EEG is a noninvasive method but has limitations of low SNR, low spatial resolution, and only up to a 100 Hz signal can be detected because it is far from the signal source (brain). 

**Figure 8:**
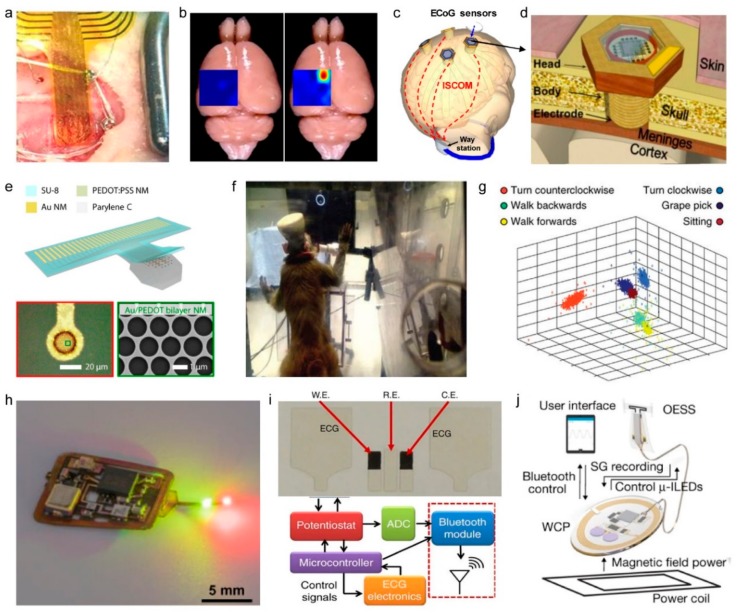
Neural interfaces (**a−g**) and physiological monitoring devices (**h−j**). (**a**) Implanted flexible ECoG electrode. A photo of the flexible electrode array placed on the left hemisphere of the brain of a Sprague-Dawley rat (Reproduced under the terms of CC BY license [141]. Copyrights 2017, the authors, Springer Nature). (**b**) Mappings of ECoG signal amplitudes from a rat brain under normal state and epilepsy state. The red area has the highest amplitude, i.e. most active under epilepsy (Reproduced under the terms of CC BY license [141]. Copyrights 2017, the authors, Springer Nature). (**c**) Schematic illustration of distributed wireless ECoG recording systems with intra-skin communication (ISCOM) (Reproduced under the terms of CC BY license [171]. Copyright 2018, MDPI AG. e) Reproduced under the terms of CC BY-NC license [144]. Copyright 2018, the authors, American Association for the Advancement of Science). (**d**) Schematic illustration of a wireless ECoG sensor (Reproduced under the terms of CC BY license [171]. Copyright 2018, MDPI AG. e) Reproduced under the terms of CC BY-NC license [144]. Copyright 2018, the authors, American Association for the Advancement of Science). (**e**) Top: Device schematic of the 32-channel Au/PEDOT: PSS nanomesh MEA. Bottom: Microscope image of a Au/PEDOT: PSS bilayer-nanomesh microelectrode (left) and a SEM image of a zoomed-in region of the microelectrode (right). (**f**) Monkey with a wireless system performing a task using pure brain control (Reproduced with permission [145]. Copyright 2014, Springer Nature). (**g**) first three principal components of neural activity for six different behaviors in a monkey. Principal-component analysis data were used for support vector machine classification (Reproduced with permission [145]. Copyright 2014, Springer Nature). (**h**) Integrated wireless, battery-free oximeters in operation mode with illuminating LEDs (Reproduced under the terms of CC BY-NC license [15]. Copyrights 2019, the authors, American Association for the Advancement of Science). (**i**) Photograph of an integrated sensing patch and a block diagram of the wireless readout circuit (Reproduced under the terms of CC BY license [146]. Copyright 2016, Springer Nature). (**j**) The platform consists of an optoelectronic stimulation and sensing (OESS) module and a low-modulus, stretchable strain gauge (SG) with integrated LEDs that wraps around the bladder to monitor changes in its volume and to provide optogenetic stimulation to the neurons that innervate the bladder (Reproduced with permission [147], Copyright 2019, Springer Nature).

The neural interfaces of electrocorticography (ECoG) increase signal quality and acuity by opening the skull and attaching the sensor directly to the cortical surface [11,170]. As neural interfaces are implanted into the skull via ECoG, wireless technologies are beginning to be integrated into neural interfaces in an effort to continuously receive powers and to obtain signals between implanted devices and external computers or measurement instruments. Xie et al. connected the ECoG sensor to a commercially available electrophysiology logic chip and implanted the chip on the surface of the mouse brain, as shown in Figure 8a. The ECoG sensor transferred the mouse’s electrocorticographic data to a smartphone via Bluetooth module. The ECoG sensor was capable of distinguishing the normal state and the epilepsy state by mapping the brain with 32 microelectrodes (Figure 8b) [141]. Chang et al. developed a wireless ECoG system that transferred signals from ECoG sensors to a neck-mounted receiver using intra-skin communication, which uses the skin as a conductive pathway for wireless data transmission (Figure 8c,d) [171]. Intra-skin communication enabled the transfer of data with low power (0.2 mW) and simultaneously received signals from 16 ECoG sensors to one receiver [172].

ECoG sensing also detects electrical activity in the brain in the form of field potentials on cortical surfaces, similar to EEG. Understanding and analyzing the working principles of the nervous system is an important goal of modern biomedical research. In order to detect several kHz of action potentials on a single-neuron scale, recording electrodes should be made smaller, to the level of neurons. Qiang et al. fabricated a neural interface of mesh structure using PEDOT:PSS, with a diameter of approximately 20 µm, as a tissue-interfacing electrode, as shown in Figure 8e. The neural interface was used to observe the action potential of the brain surface. The measured signals were wirelessly transferred by a custom-made ultra-wideband circuit [144].

Since brains possess three-dimensional neural networks, penetrative neural interfaces that can record the deep-brain region were also required [9]. Therefore, many microelectrode arrays (MEA), which penetrate brain tissue, were developed, including Utah arrays and Michigan probes, and studies have been conducted that integrated such implantable devices with wireless systems [173]. Schwarz et al. designed an array containing 448 microelectrodes to wirelessly measure the brain signals of a monkey (Figure 8f) and to analyze the signals according to the behavior of the monkey. As shown in Figure 8g, wireless brain mapping allowed the identification of signals that depended on behaviors such as walking, climbing, or picking grapes [145].

### 4.4. Physiological Monitoring Devices

In addition to neural interfaces for the analysis of the brain’s electrophysiological signals, it is very important to monitor the body’s various physiological signals in real time [174]. For example, the monitoring of oxygen concentration in localized tissues is useful for diagnosing interactions between oxygen dynamics and neural activity, identifying tumors, and wound healing. Cardiac monitoring is essential for the diagnosis of heart arrhythmia and atrial fibrillation. By identifying the disorders of the heart rhythm, symptoms such as fainting and stroke can be prevented [125]. In addition, respiration monitoring has important implications for fitness monitoring and for prevention of situations such as and sleep apnea. Accordingly, wireless sensor systems have been developed to measure various physiological signals. Zhang et al. constructed an optical sensing system of tissue oxygen consisting of LEDs and a photodiode for local tissue oximetry (Figure 8h). The sensor was applied to measure oxygen saturation in the deep brain tissue of a rat. The device was powered through resonance coupling to drive wirelessly during inserted in tissues. In addition, the sensor system is implemented to transmit the data outside through infrared communication [15].

Devices have been developed to enable comprehensive diagnostics through the integrated sensing of complex physiological parameters. Imani et al. developed a wearable multiplex sensor system that can simultaneously perform electrocardiography and lactate sensing by integrated fitness monitoring (Figure 8i). The device can be attached to the skin and transmitted multiple data using a Bluetooth module powered by a battery [146].

Also, recent research has been conducted to integrate the function of physiological monitoring and modulation. Mickie et al. developed the wireless device system that monitors the strain and enables a closed-loop modulation of bladder function (Figure 8j). For the modulation, optogenetic stimulation is arranged using LEDs. The strain data was transferred external to the body by a Bluetooth module powered by resonance coupling [147].

### 4.5. Retinal Prostheses

**Figure 9:**
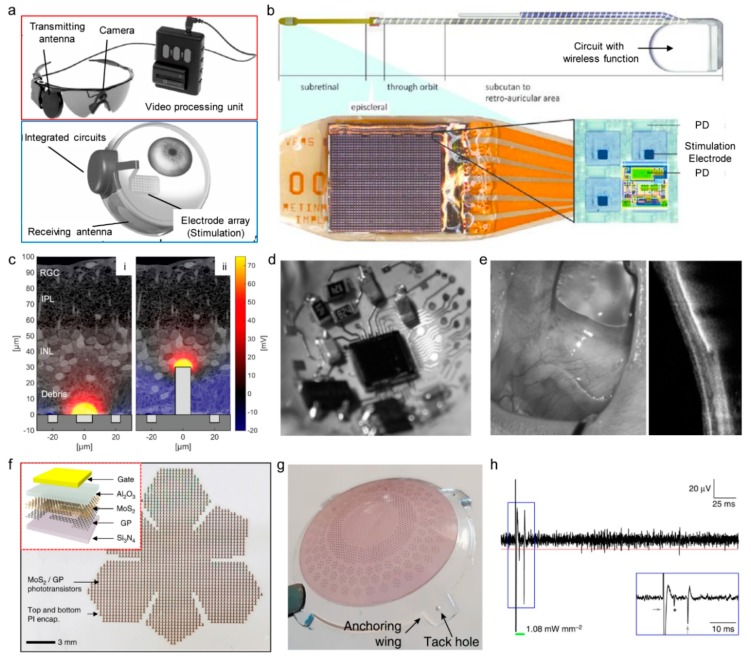
Retinal prostheses. *(***a**) Schematic illustration of a neural prosthesis system with an external camera and internal electrode arrays (Argus II System) (Reproduced under the terms of CC BY-NC-ND license []. Copyright 2016, American Academy of Ophthalmology). (**b**) Schematic illustration of a fully-internal neural prosthesis system (Alpha-IMS system). PD: photodetector (Reproduced with permission [179]. Copyright 2014, Oxford University Press). (**c**) Motivation for a 3D subretinal prosthesis. Degenerated RCS retinas exhibit near-complete loss of photoreceptors at P120, which are replaced by debris (≈ 30 *μ*m thick), separating the inner nuclear layer (INL) from RPE. Such separation between the subretinal electrodes and target neurons in INL reduces stimulation efficacy (i). Pillar electrodes (30 µm tall) can bypass debris and more effectively deliver electric field to the target cells (ii) (Reproduced with permission [152]. Copyright 2014, IOP Publishing). (**d**) Fabricated liquid crystal polymer-based retinal prosthesis with three-dimensionally integrated circuit (Reproduced with permission [148]. Copyright 2015, IEEE). (**e**) The retinal prosthesis after one year of implantation showing well recovered ocular tissues (left) and showing no adverse effect such as retinal inflammation by optical coherence tomography (right) (Reproduced with permission [148]. Copyright 2015, IEEE). (**f**) The optical camera image of the phototransistor array with a truncated icosahedron design on a planar substrate. Inset shows a schematic illustration of the device structure (Reproduced under the terms of CC BY license [149]. Copyright 2017, Springer Nature). (**g**) Photograph of lens-shaped retinal prostheses. Four anchoring wings with holes are present for attaching the prosthesis with retinal tacks (Reproduced under the terms of CC BY license [182]. Copyright 2018, Springer Nature). (**h**) Representative single-sweep recording from a retinal ganglion cell over PDMS–photovoltaic interface upon 10-ms illumination at 1081.7 µW mm^−^^2^. The red dotted line is the threshold set for spike detection. The green bar represents the light pulse. The blue insert shows a magnification of the period around the light pulse. The asterisk indicates the over-threshold spike detected, while the gray arrows are the on-set and off-set stimulation artifacts (Reproduced under the terms of CC BY license [182]. Copyright 2018, Springer Nature).

Patients with poor vision have greater difficulty in recognizing objects, and the quality of life is significantly lower than for other types of patients [175]. To restore vision and to treat incurable ocular diseases, retinal prostheses have been developed. In the retina, when light enters the eye as a stimulation, photoreceptor cells convert the light signal into an electrical pulse signal that is passed through bipolar cells and retinal ganglion cells to the brain via optic nerves [176]. In visual degeneration, such as retinitis pigmentosa or macular degeneration, retinal cells lose their signal transduction or transfer function, which results in the loss of sight [123,177]. To solve visual degeneration, retinal prostheses were developed, which consist of a camera or photodetector arrays that replace the function of photoreceptor cells, neural stimulation electrodes that deliver the electrical signals from the photodetector to retinal cells, and a wireless power supply system. Retinal prostheses can be divided into two types: (1) the camera is mounted external to the body, and the camera’s signal is transmitted to the neural stimulation electrodes in the body (Figure 9a) [178]; (2) photodetector arrays and neural stimulation electrodes are directly connected inside the body (Figure 9b) [179].

Despite many previous studies of visual prostheses for treating retinal degeneration, approaches based on conventional electronic materials and manufacturing processes result in bulky and rigid device schemes that only mimic the limited functionality of retina. With the recent development of wearable electronics, studies can be performed using materials and devices with three-dimensional or stretchable form factors that can be matched to biological tissues and organs. Further integration with wireless technologies has increased user comfort and improved performance of bioelectronic devices, which makes retinal prostheses more wearable and functional [1,180,181]. Flores et al. studied the three-dimensional configuration of the stimulation electrodes of retinal prostheses, for low-impedance with a high spatial resolution (Figure 9c) [133,]. Jeong et al. fabricated a retinal prosthesis system by laminating the stimulator circuit and antenna connected to a stimulation electrode three-dimensionally with a biocompatible liquid-crystal polymer. The three-dimensional convex structure matches the curvature of the eye (Figure 9d). A year after insertion into the rabbit’s eye, there was no visible inflammation or migration, as shown in Figure 9e [148]. Choi et al. designed the photodetector arrays with hemispherical shapes, in order to structurally match the curve of the eye when inserted as a retinal prosthesis (Figure 9f). The conformal retinal prosthesis can cover a larger area of retina, which results in a wider field-of-view than the planar photodetector arrays. The signal from the hemispherical photodetector array was connected to the optic nerve of mouse to confirm that the signal from the photodetector was transmitted to the brain [149].

In the case of retinal prostheses, data transmission must be performed from an external camera to an internal stimulation circuit, and the device must be powered from an external power source. However, the existence of power transfer and data transfer systems has made the entire external and internal device system bulky, and therefore it was necessary to develop a sustainable, fully-internal system for retinal prostheses. Since photovoltaic devices can generate electrical signals by external lights, self-powered retinal prosthesis systems have been recently researched, by arranging photovoltaic cells with stimulating electrodes instead of a photodetector that requires a power supply []. Ferlauto et al. constructed a photovoltaic retinal prosthesis system with a three-dimensional structure, as shown in Figure 9g. By implanting the retinal prosthesis to the retina of blind mice ex vivo, the detection of photoelectric signals in retinal neurons was confirmed (Figure 9h) [182].

Photovoltaic retinal prostheses are battery-free systems that can remove the power source and realize fully implantable system without external power supply. However, until now, most photovoltaic cells material used for retinal prostheses is based on silicon. Silicon photovoltaic cells receive infrared light for generating electricity, so an external device is required that converts visible light signals into an infrared signal. Therefore, it is necessary to use photovoltaic cells capable of converting visible light directly to electrical signals with high efficiency.

## 5. Prospects

Many wireless sensing applications involve attachment to bodily surfaces or implantation into the body. Therefore, further research on interference and signal reliability of wireless technologies in biological systems is needed. Electromagnetic waves are greatly affected by the surrounding environment, and when signals are transmitted from inside the body to the outside, the signal transmission regime is different from that in ambient air. Also, wearable devices and wireless signals may interfere with micro- and macroscopic movements of the body and data may be easily distorted. Therefore, analysis criteria and stable wireless technologies should be researched and selected for each biological system. In the case of wireless power transfer, heat generation and biocompatibility with high RF voltage also need to be verified for each body part targeted by the application.

In addition, current wireless circuits are mostly based on bulk, rigid plastic circuit boards, the development of which has lagged behind the development of wearable sensors with a flexible and conformal form. This problem occurs since current circuit components have high integrity, but it is inevitable to use existing rigid, flat semiconductor fabrication processes as compared to low integrity, single-function sensor. In order to increase the integrity as a wearable system by assembling circuit components on a stretchable substrate and structurally engineering them using stretchable electrodes, it is necessary to develop fabrication processes for stretchable materials and devices that can replace the existing semiconductor fabrication processes.

## 6. Conclusions

In the last several decades, we have observed advances in wearable sensors based on wireless technology and wireless device systems as specific applications. The application of wireless functions has confirmed the feasibility of attachable and implantable wearable electronics with real-time and continuous monitoring capabilities, which has been one of the ultimate goals of wearable sensors. The integrity of wireless systems on devices will continue to improve our lives. Although this review focused on wireless systems with an emphasis on healthcare devices worn by humans, this technology can be applied to robotics or the monitoring of extreme environments.

Although there are some challenges such as signal distortion or low form factor of wireless circuitries discussed above, various research groups are currently collaborating on ways of solving these problems, and these efforts may result in the development of wireless sensors and electronics that blur the boundary between humans and electronics.

## Figures and Tables

**Table 1:** Comparison of wireless data communication technologies used in wireless sensor applications.

	RFID	Bluetooth	NFC	Zigbee	Resonant Antenna	Optical
Transceiver type/Power source	Passive transponder, Battery-free or battery-assisted	Active radio, Battery power	Passive transponder, Battery-free or -assisted	Active radio, Battery power	Passive transponder, Battery-free or -assisted	Active radio, Battery power
Operating frequency	120~140 kHz 13.56 MHz 868~956 MHz	2.4~5 GHz	13.56 MHz	868 MHz, 915 MHz, 2.4 GHz	Depending on the type of antenna	30 kHz ~300 GHz
Data rate	100 kb/s	24 MB/s	424 kb/s	250 kb/s	100 kb/s	1 Gb/s
Working ranges	15 m, (frequency dependent)	10~100 m	5 cm	10~100 m	1 m (typically) (frequency dependent)	10 m
Power consumption	Very low (passive)	Medium	Low (in passive case)	Medium	Very low (in passive case)	Relatively high
Continuous monitoring	△ Portable, Smartphone-tagged	○	△ Portable, Smartphone-tagged	○	△ Network analyzer or amplifier required	○
Sensor network	○	○	○	○	○	○
Advantage	low power consumption	High data rate, Wide range	Low power consumption High security	Low power consumption Wide range	Low power consumption to transfer signal	Radiation-free No bandwidth regulation High data rate
Weakness	Low data rate Short range	High power consumption Poor security	Very short range	Lowutilization	Low data rate Short range	Light interference no passes through walls
Reference	[18,42,43,44,45,46,47]	[48,49,50,51,52]	[53,54,55,56]	[57,58,59]	[60,61,62,63]	[15,64,65,66]

**Table 2:** Summary of the reviewed works in various applications.

Ref.	Application	Sensing Type	Target Analytes	Power Supply	Data Communication	Features
[137]	Electronic skin	Electrophysiological/Physical	ECG/Body strain	Battery	Bluetooth	Display integrated
[138]	Electronic skin	Physical (triboelectric sensing)	Touch	TENG	Wi-Fi	Self-powered
[53]	Electronic skin	Electrophysiological/Physical	ECG/Skin Temperature, Hydration	Smartphone (passive type)	NFC	-
[139]	Electronic skin	Physical/Chemical	Sweat rate/pH, Lactate, Glucose, Chloride	Smartphone (passive type)	NFC	Microfluidic platform
[140]	Smart contact lens	Physical	Intraocular pressure	No power required	Resonant antenna	-
[45]	Smart contact lens	Physical/Chemical	Intraocular pressure/Glucose	No power required	Resonant antenna	Fully transparent
[3]	Smart contact lens	Chemical	Glucose	Inductive coupling	LED output	LED integrated
[141]	Neural interface	Electrophysiological	ECoG	Battery	Bluetooth	-
[142,143]	Neural interface	Electrophysiological	ECoG	Battery	Intraskin communication	-
[144]	Neural interface	Electrophysiological	Neural signals	N/A	RF antenna (Ultrawideband)	-
[145]	Neural interface	Electrophysiological	Neural signals	Battery	RF antenna (ISM band)	-
[15]	Physiology monitoring	Optical	Tissue oxygen saturation	Resonant inductive coupling	IR	-
[146]	Physiology monitoring	Electrophysiological, Chemical	ECG, Lactate	Battery	Bluetooth	-
[147]	Physiology monitoring	Physical	Bladder strain	Resonant inductive coupling	Bluetooth	Stimulation function integrated
[148]	Retinal prosthesis	Optical (camera)	Light	Battery	RF antenna	-
[149]	Retinal prosthesis	Optical (photodetector array)	Light	Battery	N/A	-
[150,151]	Retinal prosthesis	Optical (photovoltaic sensing)	Light	Photovoltaics	N/A	-
